# Experiences of and recommendations on clinical trial design in Alzheimer’s disease from the participant’s point of view: a mixed-methods study in two clinical trial centers in the Netherlands

**DOI:** 10.1186/s13195-023-01190-0

**Published:** 2023-04-04

**Authors:** Lois Ottenhoff, Everard G. B. Vijverberg, Leonie N. C. Visser, Merike Verijp, Niels D. Prins, Wiesje M. Van der Flier, Sietske A. M. Sikkes

**Affiliations:** 1 Alzheimer Center Amsterdam, Neurology, Vrije Universiteit Amsterdam, Amsterdam UMC, location VUmc, De Boelelaan 1118, 1081 HZ Amsterdam, The Netherlands; 2 Brain Research Center Amsterdam, Amsterdam, The Netherlands; 3 Amsterdam Neuroscience, Neurodegeneration, Amsterdam, the Netherlands; 4 Department of Medical Psychology, Amsterdam UMC, University of Amsterdam, Amsterdam, the Netherlands; Amsterdam Public Health Research Institute, Quality of Care, Amsterdam, the Netherlands, Amsterdam, The Netherlands; 5 Center for Alzheimer Research, Division of Clinical Geriatrics, Department of Neurobiology, Care Sciences and Society (NVS), Karolinska Institutet, Solna, Sweden; 6 Epidemiology and Data Science, Vrije Universiteit Amsterdam, Amsterdam UMC, location VUmc, Amsterdam, The Netherlands; 7 Department of Clinical, Neuro and Developmental Psychology, Faculty of Behavioural and Movement Sciences, VU University, Amsterdam, The Netherlands

**Keywords:** Alzheimer’s disease, Mild cognitive impairment, Engagement in research trials, Patients, Qualitative research, Quantitative research, Alzheimer’s disease clinical trials, Patient preferences

## Abstract

**Introduction:**

In the context of the development of pharmaceutical interventions, expectations and experiences of participants are essential. Their insights may be particularly helpful to address the challenges of recruiting and retaining participants for Alzheimer’s disease (AD) clinical trials. We examined clinical trial participants’ experiences to optimize trial design in Alzheimer’s disease (AD).

**Method:**

In this mixed-methods study, we included adults who participated in sponsor-initiated AD trials at Brain Research Center, a clinical trial organization in the Netherlands. Participants (*N* = 71, age 69 ± 6.5, 54%F, 19 cognitively normal (CN), 19 mild cognitive impairment (MCI), and 33 AD dementia) first completed an online survey. Diagnostic group differences were investigated using chi-square tests or one-way ANOVAs. Next, a subsample (*N* = 12; 8 = CN, 4 = MCI) participated in focus groups to gain in-depth insight into their opinions on optimizing trial design from a participants’ point of view. Audio recordings from focus group interviews were transcribed verbatim and analyzed by thematic content analysis by two independent researchers.

**Results:**

Most reported motives for enrolment included “to benefit future generations” (89%), followed by “for science” (66%) and “better monitoring” (42%). Frequent suggestions for increasing willingness to participate included a smaller chance to receive placebo (*n* = 38, 54%), shorter travel times (*n* = 27, 38%), and sharing individual results of different assessments (*n* = 57, 80%), as well as receiving trial results (*n* = 52, 73). Highest visual analogue burden scores (0–100) were found for the lumbar puncture (M = 47.2, SD = 38.2) and cognitive assessments (M = 27.2, SD = 25.7). Results did not differ between diagnostic groups, nor between patient and caregiver participants (all *p*-values>.05). Two additional themes emerged from the focus groups: “trial design,” such as follow-up visit(s) after participating, and “trial center,” including the relevance of a professional and empathic staff.

**Conclusion:**

Relevant factors include expectation management and careful planning of high-burden assessments, provision of individual feedback, and prioritizing professionalism and empathy throughout conduct of the trial. Our findings provide insight into participants’ priorities to increase willingness to participate and can be used to optimize trial success.

**Supplementary Information:**

The online version contains supplementary material available at 10.1186/s13195-023-01190-0.

## Background

Alzheimer’s disease (AD) is among the largest health care challenges of our century, with almost 50 million people diagnosed with AD worldwide [1, 2]. Therefore, new ways to prevent, delay, or treat AD are urgently needed. Clinical trials are, in line with the growing body of evidence suggesting that underlying pathology precedes the onset of AD dementia, shifting towards including people who are in the prodromal and preclinical phases of AD [3].

Recruitment and retention of participants have been a major bottleneck in conducting clinical trials [4, 5]. Finding sufficient participants who remain in the trial until the end is essential for its success. Several studies have explored motivations and challenges for clinical trial enrollment for people with mild cognitive impairment (MCI) due to AD [6–8], and cognitively normal (CN) participants with a high risk of AD [9]. The challenges of recruitment for AD clinical trials include fear of side-effects of medications [10], the chance of getting placebo [10], fear of invasive procedures [10], requirement of a study partner [9], long study durations [11], and large number of visits and logistic concerns [10–12]. However, much of the relevant literature is based on the perceptions of health care professionals, or on hypothetical clinical trials rather than the perceptions of the actual participants, that is both patients and their caregivers [6, 11–14]. Also, it is important to take into account differences between diagnostic groups. Incorporating participants’ priorities, concerns, and suggestions into clinical trial design could be highly beneficial for the recruitment and retention of trial participants, and consequently the development of the design of intervention studies [15, 16].

In this study, the experiences of participants in AD clinical trials are taken as a starting point to provide insight into elements of trial design that are important to increase willingness to participate. Specifically, we aimed to examine their motivations for trial participation, experienced burden of trial features, preferred frequency of visits, and suggestions for stimulating recruitment and retention of participants. Furthermore, we examined differences between diagnostic groups regarding their views on trial design.

## Method

### Design

**Fig. 1:**
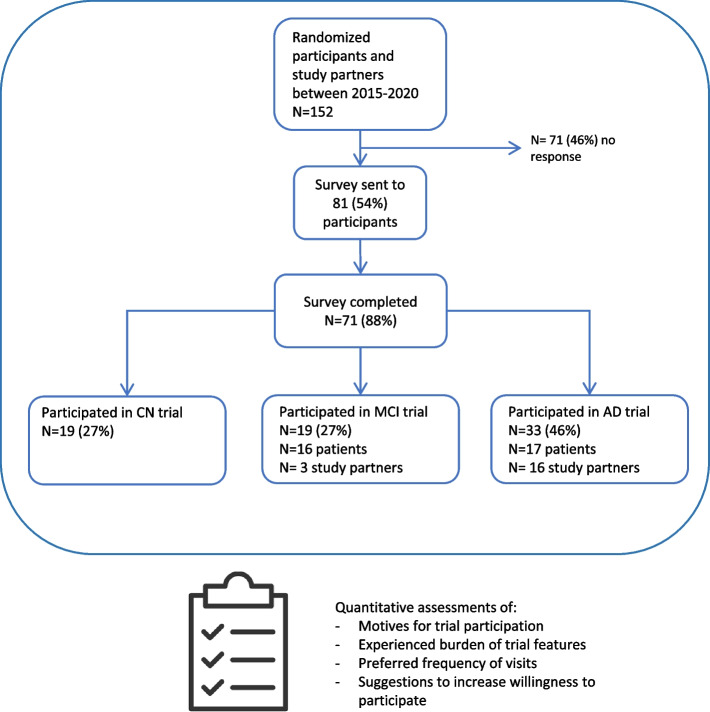
Project overview. This figure shows the steps of the survey respondents. Motives, challenges, and experiences of trial participants were investigated using the survey. Then, the answers were used as input for the focus groups to gain in-depth insight into views with regard to trial design. Abbreviation: AD, Alzheimer’s disease; MCI, mild cognitive impairment; CN, cognitively normal participants

We used a mixed-methods study design in which the quantitative data collection, i.e., a survey among trial participants and study partners, informed the qualitative collection, i.e., focus groups with trial participants [17]. Participants were recruited via Brain Research Center (BRC), a specialized clinical trial organization in central nervous system disorders in two locations in the Netherlands: one in Amsterdam and one in Den Bosch. We included participants and their study partners who participated in 11 international sponsor-initiated trials between 2015 and 2020 at one of two different BRC sites in the Netherlands. These included participants with AD dementia, participants with MCI due to AD or cognitively normal (CN) participants who were amyloid positive and/or APOE E4 carriers. First, we asked the 152 eligible trial participants and/or their study partners if they were interested to participate in a survey that was distributed online. Out of these, 81 (54%) were interested in participating and were sent an email that included a link to the survey (Fig. 1: Survey overview). Of these 81 participants, 71 (88%) completed the survey. Second, we asked these participants if they were interested in participating in an additional focus group; 26 (37%) participants indicated to be interested, and we used purposive sampling to select 12 focus group participants. The study protocol was approved by the Medical Ethical Committee of the Amsterdam UMC, location VU. Participants in the survey provided online digital consent, and focus group participants provided additional written informed consent.

### Measurements

#### Survey

The content of the survey was based on previous studies focusing on challenges and motivation for participation in AD prevention trials [6–19] and supplemented with additional topics based on the aims of this study. The complete survey is included in Additional file 1. The survey comprised the following domains: demographics, rationale, and motivations for trial participation; experienced participant burden; preferred frequency of visits; and suggestions for stimulating participation. Additional items asked participants to report their diagnosis and in which trial they participated, to check the research consensus diagnoses. The online survey was distributed via an anonymous link through Castor, an online survey system (castoredc.com).

#### Focus groups

Next, we conducted focus groups to gain in-depth insight into the views of participants with regard to participation in clinical trials and their opinions on the optimal trial design to find out what aspects trial participants think are most important. Three focus groups were needed to reach data saturation, i.e., no new themes emerged from the third focus group in comparison to the data obtained from the first two. One focus group included 4 participants with MCI due to AD currently participating in a trial, and two focus groups included in total 8 CN participants, who had previously participated in a trial. Nominal group and Participatory Learning and Action (PLA) techniques were used to inspire the design of the focus groups [20, 21]. Each focus group consisted of two parts. First, a brainstorming session to generate bottom-up, inductive data of positive and negative aspects of their trial participation. Participants were given 10 min to generate a list of all issues they considered relevant, in response to the question “What are important negative and/or positive factors or aspects as experienced during your participation in a trial?.” After 10 min, a round-robin approach was used to collate ideas, that is, each participant in turn was asked to read one item off their list. When each participant had mentioned a positive or negative aspect, the same process was repeated until each participant’s list of items was exhausted. Participants were asked not to repeat an item already given by a participant. After a final, comprehensive list of positive and negative aspects was established, each participant was then asked to ascribe a score between 0 (no importance) and 10 (great importance) to each of the trial aspects. Finally, they were asked to identify the five items that they considered most important.

The second part of the focus groups was centered on discussing and reflecting on the optimal trial design or design features. Here, the participants were instructed to vote which trial design they preferred out of three possible designs, which were different in terms of study duration, placebo chances, and frequency of visits and follow-up visits, features that we selected based on responses of the survey and of two actual trials. They were first asked to select one trial design they preferred and explain to the group why. They were then asked to divide 10 votes over the three trial designs, appointing more votes to the study they would most likely want to participate in, and explain why. The process of voting and justifying/explaining the rationale behind their votes was intended to elicit a discussion on all relevant features of trial design, in a guided manner. Each focus group lasted approximately 2 h and was audio-recorded and transcribed verbatim. All data were anonymized.

### Data analysis

Data from completed surveys were used in analyses (5 were not completed). Descriptive statistics were used to report participant characteristics. One-way ANOVA, *T*-tests, and chi-square tests were used to determine whether participants’ motivations, experienced burden, and frequency of visits differed between the diagnostic groups, and between patient and caregiver participants. IBM SPSS Statistics, version 22, was used, and *p*-values less than .05 were considered statistically significant.

All positive and negative aspects of AD clinical trials that were assembled during the first part of the focus groups were combined to one comprehensive list, including the summed importance scores with a potential range from 0 to 120 over the 12 participants, and the frequency of being included in participants’ top 5. Conventional content analysis [22] was performed on the transcripts of the second part of the focus groups to establish the most optimal clinical trial design features emerging from the data. All transcripts were independently coded by two researchers (L.O. and M.V.). As a first step, the transcripts were read and an initial list of optimal features or themes was generated by L.O. Next, the researchers independently categorized all relevant statements that came up in the discussion, i.e., they assigned each statement to a design feature or an overall theme. Codes were then compared and discussed until consensus was reached, resulting in a final coding framework.

## Results

### Survey

#### Participant characteristics

Seventy-one participants completed the survey, of which 19 participated in a trial for CN, 19 in an AD trial in stage MCI, and 33 in an AD dementia clinical trial (Table 1). One participant self-reported a diagnosis of MCI, but he participated in a trial for CN participants with high risk of AD. In analyses assessing attitudes towards trial participation, this patient was included as part of the CN group. The majority of survey respondents took part in a single trial (*n* = 45, 63.3%), and in the MCI group, participation in multiple trials was more common (*n* = 10/19, *p* = .011).

**Table 1:** Characteristics of survey respondents

Characteristics	All survey respondents	Alzheimer dementia	Mild cognitive impairment	Cognitively normal	*p*-value
*N*	71	33	19	19	
Age, mean (±SD)	68.7 (6.5) Range = 51–82	67.6 (7.9) Range = 51–81	68.8 (8.8) Range = 53–82	69.9 (4.1) ^b^ Range = 63–82	.56
Female, *n* (%)	38 (54)	21 (64)	10 (53)	7 (37)	.17
Education level, *n* (%)					.18
Up to secondary school completed	23 (32)	13 (39)	6 (32)	4 (21)	
Vocational training, diploma	11 (16)	5 (15)	5 (26)	1 (5)	
University degree	37 (52)	15 (46)	8 (42)	14 (74)	
Participated in a number of clinical trials					
1/2/3, *n*	45/17/ 9	19/9/5	9/6/4	17/2/0	.02^
Completed participation in clinical trial^a^	32 (45%)	18 (55%)	7 (37%)	7 (37%)	.15
Study partner	19 (7%)	16 (49%)	3 (16%)	0 (0%)	<.01

Data are represented as mean (SD) or as *n* (%)

^a^Data were available for *n*=66

^b^Data available for *n*=18

^Tested for 1 versus multiple trial participations

#### Rationale and motives

Most participants reported initially enrolling for the benefit of future generations (*n* = 63, 89%), followed by “for science” (*n* = 47, 66%) and “better monitoring” (*n* = 30, 42%) (Table 2)*.* The majority of the participants (*n* = 51, 72%) were “very” to “extremely” satisfied about their participation, and the large majority (*n* = 63, 89%) indicated they would be likely to enroll in another AD clinical trial. When participants were asked whether they considered to stop during their participation, most participants (*n* = 67, 94%) never thought of quitting. Still, four of them did, because of the following reasons: side effect(s) from the lumbar puncture (*n* = 1), experienced decline in memory (*n* = 1), the role of the pharmaceutical company (*n* = 1), and general experienced burden (*n* = 1). No differences were observed between diagnostic groups, nor between patient and caregiver participants (all *p* > .05)

**Table 2:** Summary table of answers to the multiresponse question “what was the most important reason to participate in a clinical trial?”, stratified per diagnostic group

Most important reason to participate in a clinical trial	Overall		Alzheimer’s disease		Mild Cognitive impairment		Cognitively normal	
	N	% (n_**total**_=71)	n	% (n_**AD**_=33)	n	% (n_**MCI**_=19)	n	% (n_**CN**_=19)
For the future generation	63	88.7%	28	94.3%	17	98.5%	18	94.7%
For science	47	66.2%	21	63.6%	15	78.9%	11	57.9%
I think I will be monitored better	30	42.3%	14	91.5%	9	47.4%	7	36.8%
I find it interesting	22	31.0%	12	36.4%	162	39.7%	5	26.3%
My doctor recommended it	7	9.9%	5	15.2%	2	10.5%	0	0.0%
I think it is the best treatment	7	9.9%	5	15.2%	2	10.5%	0	0.0%
To receive better care	7	9.9%	4	12.1%	2	10.5%	1	5.3%
It is a useful time to spend the day	2	2.8%	2	6.1%	0	0.0%	0	0%

#### Suggestions for increasing willingness to participate

Suggestions to improve willingness to participate were investigated by two multiresponse questions: “What would make participation easier for you or others” and “What factors are important to consider when enrolling again?”. The most frequently selected reasons to the first question were a smaller chance of receiving placebo (*n* = 38, 54%) and shorter travel times (*n* = 27, 38%) (Table 3). Most reported factors considered important when enrolling again included receiving individual results of different assessments (*n* = 57, 80%) and receiving research results of the trial (*n* = 52, 73%) (Table 4). The least frequently selected reasons for future enrolment were receiving payment (*n* = 4, 6%) and privacy (*n* = 3, 4%).

**Table 3:** Summary table of answers to the multiresponse question “what would make participation easier for you or others?”, stratified per diagnostic group

What would make participation easier for you or others?	Overall		Alzheimer’s disease		Mild Cognitive impairment		Cognitively normal	
	N	% (n_**total**_=71)	n	% (n_**AD**_=33)	n	% (n_**MCI**_=19)	n	% (n_**CN**_=19)
Less chance to receive placebo	38	53.5%	18	54.5%	13	68.4%	7	36.8%
Shorter travel time	27	38.0%	14	42.4%	6	31.6%	7	36.8%
Less frequent lumbar puncture	22	31.0%	12	36.4%	4	21.1%	6	31.6%
Home visits	12	16.9%	9	27.3%	2	10.5%	1	5.3%
Less frequent visits to center	6	8.5%	5	15.2%	1	5.3%	0	0.0%
Study partner burden	9	12.7%	4	12.1%	1	5.3%	4	21.1%
Less frequent MRI scan	8	11.3%	3	9.1%	1	5.3%	4	21.1%
Less frequent PET scan	6	8.5%	3	9.1%	1	5.3%	2	10.5%
More compensation	5	7.0%	1	3.0%	4	21.1%	0	0%
Less frequent memory assessments	4	5.6%	2	6.1%	1	5.3%	1	5.3%
Less frequent depression assessments	2	2.8%	0	0%	2	10.5%	0	0%

**Table 4:** Summary table of answers to the multiresponse question “what are the most important factors when considering to enroll again?”, stratified per diagnostic group

What are the most important factors when considering to enrol again?	Overall		Alzheimer’s disease		Mild Cognitive impairment		Cognitively normal	
	N	% (n_**total**_=71)	n	% (n_**AD**_=33)	n	% (n_**MCI**_=19)	n	% (n_**CN**_=19)
Sharing personal results	57	80.3%	24	72.7%	16	84.2%	17	89.5%
Sharing research results	52	73.2%	22	66.7%	16	84.2%	14	73.7%
Possibility to enrol in a new trial	45	63.4%	24	72.7%	13	68.4%	8	42.1%
The same specialist each visit	41	57.7%	18	54.5%	13	68.4%	10	52.6%
Reputation research center	34	47.9%	16	48.5%	10	52.6%	8	42.1%
Number of visits to center per month	11	15.5%	7	21.2%	4	21.1%	0	0%
Side effects	10	14.1%	3	9.1%	4	21.1%	3	15.8%
Distance to study center	8	11.3%	4	12.1%	3	15.8%	1	5.3%
Chance to receive placebo	8	11.3%	4	12.1%	4	21.1%	0	0%
To receive payment	4	5.6%	2	6.1%	2	10.5%	0	0%
Duration of study visit	4	5.6%	3	9.1%	1	5.3%	0	0%
Privacy	3	4.2%	1	3.0%	2	10.5%	0	0%

#### Preferred frequency of visits and trial duration

Once per month was chosen most often as the preferred visit schedule to the center (*n* = 43, 61%). Participants indicated different preferred frequency of visits for the different assessments. Once per month was most frequently selected, for blood tests (*n*=33, 47%), electrocardiogram (ECG) (*n*=30, 42%), and the neurological assessments (*n*=30, 42%). Once per 3 months was most frequently selected for depression (*n*=36, 51%), quality of life (QoL) (*n*=36, 49%) and instrumental activities of daily living (IADL) (*n*=35, 49%), and questionnaires. This was also the most selected preference for the neuropsychological assessments (*n*=34, 48%) and MRI scan (*n*=27, 38%) frequency. Once per year was most frequently chosen as the optimal frequency for the lumbar puncture (*n*=47, 66%) and PET scan (*n*=33, 47%). The preferred trial duration of a clinical trial was on average 3.8 years (SD=2.7), with a wide range (1–10 years). A one-way ANOVA revealed that there was a significant difference in preferred trial duration between the three diagnostic groups (*F*(2,68)=9.4, *p* = <.001). Post hoc comparisons using the Tukey HSD test indicated that participants from the CN group preferred a longer trial duration (*M* = 5.8, SD=2.6) compared to participants with AD dementia (*M* = 2.8, SD = 1.9, *p*<.001) and MCI (*M*=3.5, SD=3.1, *p*=0.012).

#### Patient burden

**Fig. 2:**
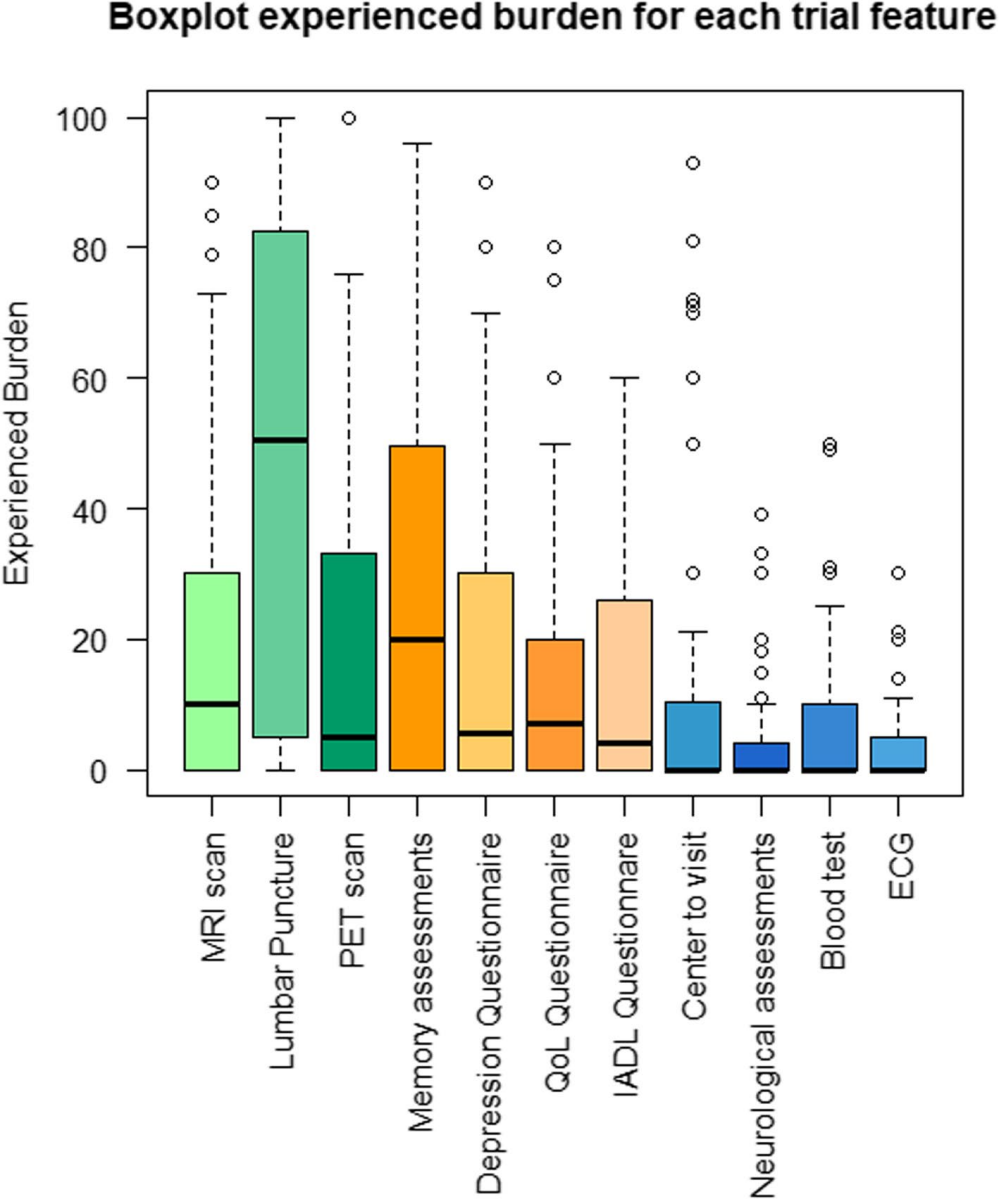
The experienced intensity of trial features

Participants rated the experienced burden of various trial assessments on a scale of 0 (no burden) to 100 (high burden), as seen in Fig. 2. Highest burden scores were observed for the lumbar puncture (*M* = 47.2, SD = 38.2), followed by cognitive assessments (*M* = 27.2, SD = 25.7) and the PET scan (*M* = 19.3, SD = 25.7). Of note, we observed a high variety in scores, and this variety was not dependent on syndrome diagnosis (*p* > .05), or being a patient or caregiver participant (*p* > .05).

### Focus groups

#### Positive and negative aspects of participating

Across the three focus groups, a comprehensive list of 18 negative and 19 positive aspects of participating in a trial was reported by the participants (Table 5). Lack of communication about results during or after trial participation, the lumbar puncture, and cognitive assessments were most often ranked as the most important negative experienced factors.

**Table 5:** Negative and positive experienced aspects of focus groups participants, including ranking every mentioned positive and negative aspects from 0 (not important) to 10 (very important)

Experienced negative aspects	Summed score	Frequency top 5	Experienced positive aspects	Summed score	Frequency top 5
Communication test results during or after participating	75/120	7/12	Empathy employees research center	107/120	8/12
Lumbar puncture	75/120	5/12	Contribute to a possible cure for Alzheimer’s disease	74/120	6/12
Cognitive assessments	70/120	4/12	Personal treatment	68/120	1/12
Communication regarding the result of genetic testing/diagnosis	70/120	3/12	Professionalism of research team	66/120	6/12
Study stopped without result	62/120	2/12	A hearty greeting of reception	65/120	4/12
Appreciation pharmaceutical company	41/120	1/12	More time/attention than in hospital	57/120	5/12
MRI scan	38/120	3/12	Provide lunch	49/120	1/12
Results of neuropsychological assessments are confronting	37/120	1/12	Keep track of brain condition	47/120	2/12
Study partner burden	36/120	3/12	Attention physical condition	46/120	4/12
Little interest in motivation of participants	34/120	1/12	Scientific approach to research	46/120	2/12
Lack of follow-up measurements after and at the end of the study	26/120	3/12	Atmosphere research location	46/120	0/12
Lack of empathy of staff outside of research center	25/120	1/12	Supervision and guidance research team	44/120	1/12
PET scan	21/120	1/12	Neuropsychological assessments	43/120	0/12
Travel distance to research center	21/120	3/12	Study partner involved	36/120	1/12
Not working devices	19/120	0/12	Frequency of visits	28/120	0/12
(unannounced) Changes in research personnel	18/120	2/12	Feedback abnormal results	26/120	3/12
Temperature research center	15/120	0/12	The distance from the study site	25/120	0/12
Starting too early	11/120	3/12	No pressure, always possibility to stop participation	20/120	0/12
			No hierarchy	19/120	4/12

*Notes*: Score: all aspects were scored by participants on a 0–10 scale, with summed scores thus ranging from 0 to 120 with a total of 12 participants (if the aspect was mentioned in each focus group)

Frequency top 5: how often participants identified the aspect as most important when they had to select the five items that they considered most important

The positive experienced aspects most often ranked in top 5 as very important were the empathy of the employees of the research center, truthfulness and professionalism of the research employees, the notion that one is contributing to a possible cure for AD, and the experience that staff has more time/attention than available during a routine hospital visit.

#### Clinical trial design

Content of the audio-recorded discussions throughout the voting process for the most optimal clinical trial revealed two main categories: features related to trial *design* and those related to the trial *center*.

##### Theme 1: trial design features

The most mentioned trial design elements that need optimization according to participants are as follows: receiving their personal test results and follow-up visit(s) after participating in a trial. Participants indicated that they would like to know their own results of different trial assessments such as cognitive assessments, lumbar puncture, and MRI scan. After having invested a lot of their time in support of a clinical trial, they feel disrespected when not receiving their personal results, as illustrated by the following quotes:

Female, 63, CN: I would like to know how I am doing after the trial. Is it going well or am I getting worse?.

Male, 70, CN: I wanted to know how I was doing since the trial stopped suddenly. Therefore, follow up visits after participating would be nice.

Participants in the preclinical AD trials found communication about amyloid status or APOE E4 carrier ship important. Most participants wished to learn their amyloid status and/ or APOE A4 risk profile, but stated that adequate communication is very important to comprehend what the results mean. Learning about their Alzheimer’s risk status had more impact for some than expected beforehand. The importance of adequate communication is highlighted in the following quote:

Male, 73, CN: Communciation is very important. In the beginning I did not understand what it meant to be an APOE E4 carrier.

Another prominent subtheme to emerge as a trial design feature was trial duration, especially combined with the chance to be randomized into the placebo group. Participants differed however in their preferences and argumentation, as illustrated below:

Female, 67, CN: Trial duration is most determative for me when participating in a trial, I would rather participate in a trial of longer duration.

Male, 73, CN: I do not like the idea that you get involved in a clinical trial for 5 years and at the end it turns out you had a placebo.

Female 76, MCI: A one year trial is perfect for me. If it then turns out the medicine is not working, I will participate in a next clinical trial.

Too many data collection points or assessments increased the burden for participants. Participants in the focus groups emphasized that they experienced high burden of the following assessments: cognitive assessments, lumbar puncture, PET scan, and MRI scan. They understood they had to undergo these procedures, but expected (more) careful planning of these high-burden assessments, i.e., only when needed. Other solutions to get blood-based biomarkers instead of lumbar puncture or a shorter duration of cognitive assessments were highly recommended. Participants explained:

Female, 63, CN: The memory assessment were very hard for me, especially the one you have to remember 15 words felt as failing. I almost started crying.

Male, 68, CN: I had a headache for one week after the lumbar puncture.

##### Theme 2: trial center features

Empathy of research staff was often mentioned as a feature of the optimal trial center. Quality and attitude of the research staff are of great importance for participants. Also, research staff being professional, warm, and approachable and making efforts to show participants they are appreciated and valued were often mentioned as highly valued. It is considered very important that study support staff should be generally aware of how study participants are feeling during the trial, and seek to minimize patient stress. Participants explained:

Female, 65, MCI: I would really appreciate being guided by the same people as much as possible.

Male, 76, CN: I was very happy with the professionalism and adequacy of the research staff.

Related to that, considering the personal motivation of trial participants was mentioned as an important factor. Participants believed that understanding the reasons and personal motivation for participation and participant expectations is crucial for retention. One participant explained:

Male, 68, CN: I thought it was striking that there was no interest in my reasons to enroll in a clinical trial. For me it was very important that the research staff knew why I participated.

Finally, both related to clinical trial design and center, the involvement, financial interests, and negative media attention of pharmaceutical companies were mentioned as important considerations to stop participation. Participants explained:

Male, 70, CN: You will not have any personal contact with the pharmaceutical company, but you have to sign all these papers for them, that feels wrong.

Male, 70, MCI: Negative publicity of the pharmaceutical company (making large financial gains) affected my willingness to participate in the trial.

## Discussion

This mixed-methods study provides insight into trial design elements important for optimal trial recruitment, participation, and retention, from the perspective of participants in AD clinical trials. Structurally receiving individualized test results and smaller chance to get placebo, as well as trial center features, were identified as motivators for trial participation.

In line with former studies, trial participants experienced neuropsychological testing and the lumbar puncture as high burden assessments [9, 23, 24]. Most trials include a broad array of neuropsychological tests, which may result in frustration and distress for the participant [25]. For trial participants, the confrontation with their cognitive struggles can be overwhelming and may result in unwillingness to participate. Shorter, more focused cognitive tests sensitive to change have been developed, such as the cognitive functional composite (CFC) [26]. Initiatives to reduce levels of anxiety and/or uncertainty with regard to the lumbar puncture procedure have been introduced [27]. Furthermore, reducing the number of lumbar punctures during the trial, for example by making use of blood-based biomarkers could provide a noninvasive and patient-friendly alternative [28].

Considering incorporating follow-up visits (follow-up or contact with the trial site after ending the trial) and sharing individualized test results with participants could improve the engagement of participants [4, 19, 29, 30]. Additional funds from study sponsors to enable longer-term clinical follow-up of participants could further enhance participants’ feeling of support [31, 32]. With regard to sharing study results, complicating (ethical) factors, such as the patient and caregivers’ coping style in receiving potentially negative results, and receiving results years after participating in the trial, might hamper disclosure [33, 34]. However, in oncology, it was demonstrated that finding out the (personal) results of a trial might make trial participation more worthwhile, as well as being highly appreciated by participants [34]. Further research is therefore recommended to study different results scenarios for the AD population.

The quality and attitude of the research staff and creating a caring and supporting environment of the trial centers were important according to the trial participants. Positive environments are created through staff being both professional as well as warm and approachable, and by making efforts to show participants that they are appreciated and valued (for example by offering them coffee or lunch). These findings are in line with previous studies on clinical trial organization, in which educated or experienced staff was found highly relevant for the execution of the growing complexity of clinical trial execution [35, 36], In our study, there was considerable variation in the positive and negative aspects of trial participation and optimal trial design. However, *all* participants mentioned the importance of a caring and supporting environment and an empathic research staff.

It is important to manage the expectations and motivations of participants at the beginning of a trial. In this way, expectations can be adjusted in time (for example, whether or not participants will receive results of their own assessments), or participants can be matched to a trial that fits their preferences with regard to study duration, or frequency of assessments that are necessary.

## Strengths and limitations

It is important to account for several study limitations when interpreting these findings. This study was limited by a homogeneous sample that consisted of predominantly highly educated and Caucasian trial participants. Also, we included participants who participated in a clinical trial and not those who did not want to participate. Furthermore, this study was conducted at two separate sites of the Brain Research Center, with participants’ motivations for enrollment possibly varying across sites. However, we explored differences in the clinical trial experiences by participants of the two sites but found no differences (data not shown). Lastly, agreeing to participate in a focus group suggests a degree of interest in medical research and interventions, creating a selection bias of highly motivated individuals, which might lead to different findings. However, previous work has not focused on participants actual participating in a clinical trial. Strengths of the current study include a more thorough understanding of which design elements can improve willingness to participate with a mixed method design. This allows researchers to design clinical trials taking into account participants’ perspectives thereby improving trial success. A particular strength is the inclusion of a substantial portion of participants who enrolled in one or more clinical AD trial. This is important, because previous research has been mainly focused on the recruitment and retention in participants without any clinical trial experience. Finally, this study did not use a hypothetical clinical trial, as is customary in focus group research, but rather took the participants’ real-life experience in actual clinical trials as starting point [6, 8, 9, 11–13].

## Conclusion

In conclusion, our findings provide insight into participants’ priorities to optimize clinical trial recruitment and ensure trial success. Recommendations of trial participants include careful planning of high burden assessments, providing individual test results and prioritizing professionalism and empathy throughout the trial. Thus, expectation management of participants at the beginning of a trial and matching of participants to a trial that fits their preferences regarding study duration and/or frequency of assessments that are required are recommended optimizations for future clinical trials, as well as for ethics committees and regulatory agencies to consider.

## Supplementary Information

**Additional file 1.** Survey OTAPA.

## Data Availability

The datasets used and/or analyzed during the current study are available from the corresponding author on reasonable request.

## References

[1] Alzheimer’s Association. What is Alzheimer’s? Available at: http://www.alz.org/alzheimers_disease_what_is_alzheimers.asp. Accessed 7 Mar 2008

[2] WHO (2015). The epidemiology and impact of dementia: current state and future trends.

[3] Cummings J, Lee G, Ritter A, Sabbagh M, Zhong K (2020). Alzheimer’s disease drug development pipeline: 2020. Alzheimers Dement (N Y).

[4] Clement C, Selman LE, Kehoe PG, Howden B, Lane JA, Horwood J (2019). Challenges to and facilitators of recruitment to an Alzheimer’s disease clinical trial: a qualitative interview study. J Alzheimers Dis.

[5] Chaudhari N, Ravi R, Gogtay NJ, Thatte UM (2020). Recruitment and retention of the participants in clinical trials: challenges and solutions. Perspect Clin Res.

[6] Lawrence V, Pickett J, Ballard C, Murray J (2014). Patient and carer views on participating in clinical trials for prodromal Alzheimer’s disease and mild cognitive impairment. Int J Geriatr Psychiatry.

[7] Sacristán JA, Aguarón A, Avendaño-Solá C, Garrido P, Carrión J, Gutiérrez A (2016). Patient involvement in clinical research: why, when, and how. Patient Prefer Adher.

[8] Cox CG, Ryan MM, Gillen DL, Grill JD (2019). A preliminary study of clinical trial enrollment decisions among people with mild cognitive impairment and their study partners. Am J Geriatr Psychiatry.

[9] Nuño MM, Gillen DL, Dosanjh KK, Brook J, Elashoff D, Ringman JM (2017). Attitudes toward clinical trials across the Alzheimer’s disease spectrum. Alzheimers Res Ther.

[10] Grill JD, Karlawish J (2010). Addressing the challenges to successful recruitment and retention in Alzheimer's disease clinical trials. Alz Res Therapy.

[11] Karlawish JH, Casarett DJ, James BD (2002). Alzheimer’s disease patients’ and caregivers’ capacity, competency, and reasons to enroll in an early-phase Alzheimer’s disease clinical trial. J Am Geriatr Soc.

[12] Karlawish J, Cary MS, Rubright J, Tenhave T (2008). How redesigning AD clinical trials might increase study partners’ willingness to participate. Neurology.

[13] Johnson FR, DiSantostefano RL, Yang JC, Reed SD, Streffer J, Levitan B (2019). Something is better than nothing: the value of active intervention in stated preferences for treatments to delay onset of Alzheimer’s disease symptoms. Value Health.

[14] Karlawish JH, Casarett D, Klocinski J, Sankar P (2001). How do AD patients and their caregivers decide whether to enroll in a clinical trial?. Neurology..

[15] Hennessy M, Hunter A, Healy P, Galvin S, Houghton C (2018). Improving trial recruitment processes: how qualitative methodologies can be used to address the top 10 research priorities identified within the PRioRiTy study. Trials..

[16] Tochel C, Smith M, Baldwin H, Gustavsson A, Ly A, Bexelius C (2019). What outcomes are important to patients with mild cognitive impairment or Alzheimer’s disease, their caregivers, and health-care professionals? A systematic review. Alzheimer's Dement.

[17] Castillo-Page L, Bodilly S, Bunton S (2012). AM last page: understanding qualitative and quantitative research paradigms in academic medicine. Acad Med.

[18] Connell CM, Boise L, Stuckey JC, Holmes SB, Hudson ML (2004). Attitudes toward the diagnosis and disclosure of dementia among family caregivers and primary care physicians. The Gerontologist.

[19] Watson J, Saunders S, Muniz Terrera G, Ritchie C, Evans A, Luz S (2019). What matters to people with memory problems, healthy volunteers and health and social care professionals in the context of developing treatment to prevent Alzheimer's dementia? A qualitative study. Health Expect.

[20] O'Reilly-de Brún M, de Brún T, O'Donnell CA, Papadakaki M, Saridaki A, Lionis C (2018). Material practices for meaningful engagement: an analysis of participatory learning and action research techniques for data generation and analysis in a health research partnership. Health Expect.

[21] McMillan SS, King M, Tully MP (2016). How to use the nominal group and Delphi techniques. Int J Clin Pharm.

[22] Hsieh HF, Shannon SE (2005). Three approaches to qualitative content analysis. Qual Health Res.

[23] Watson JL, Ryan L, Silverberg N, Cahan V, Bernard MA (2014). Obstacles and opportunities in Alzheimer’s clinical trial recruitment. Health Affairs (Project Hope).

[24] Babapour Mofrad R, Bouwman FH, Slot RER, Timmers T, van der Flier WM, Scheltens P (2017). Lumbar puncture in patients with neurologic conditions. Alzheimer's Dement.

[25] Lai JM, Hawkins KA, Gross CP, Karlawish JH (2008). J Gerontol A Biol Sci Med Sci.

[26] Jutten RJ, Harrison JE, Brunner AJ, Vreeswijk R, van Deelen RAJ, de Jong FJ (2020). The cognitive-functional composite is sensitive to clinical progression in early dementia: longitudinal findings from the catch-cog study cohort. Alzheimers Dement (N Y).

[27] Babapour Mofrad R, Fruijtier AD, Visser LNC, Hoogland N, van Dijk M, van Rossum F (2021). Lumbar puncture patient video increases knowledge and reduces uncertainty: an RCT. Alzheimers Dement (N Y).

[28] Thijssen EH, Rabinovici GD (2021). Rapid progress toward reliable blood tests for Alzheimer disease. JAMA Neurol.

[29] Pierce AL, Cox CG, Nguyen HT, Hoang D, Witbracht M, Gillen DL (2018). Participant satisfaction with learning Alzheimer disease clinical trial results. Alzheimer Dis Assoc Disord.

[30] Bardach SH, Holmes SD, Jicha GA (2018). Motivators for Alzheimer’s disease clinical trial participation. Aging Clin Exp Res.

[31] Hedman R, Hellström I, Ternestedt B-M, Hansebo G, Norberg A (2018). Sense of self in Alzheimer’s research participants. Clin Nurs Res.

[32] Geraci L, De Forrest R, Hughes M, Saenz G, Tirso R (2018). The effect of cognitive testing and feedback on older adults’ subjective age. Aging Neuropsychol Cognit.

[33] Partridge AH, Winer EP (2002). Informing clinical trial participants about study results. JAMA..

[34] South A, Joharatnam-Hogan N, Purvis C, James EC, Diaz-Montana C, Cragg WJ (2021). Testing approaches to sharing trial results with participants: the show RESPECT cluster randomised, factorial, mixed methods trial. PLoS Med.

[35] Hornung CA, Kerr J, Gluck W (2021). The competency of clinical research coordinators: the importance of education and experience. Ther Innov Regul Sci.

[36] Baer AR, Zon R, Devine S, Lyss AP (2011). The clinical research team. J Oncol Pract.

