# Supplementary material for: Experiences of and recommendations on clinical trial design in Alzheimer’s disease from the participant’s point of view: a mixed-methods study in two clinical trial centers in the Netherlands
Source: Alzheimers Res Ther. 2023 Apr 4;15:72. doi: 10.1186/s13195-023-01190-0 (PMC10071606; doi:10.1186/s13195-023-01190-0)
Supplement: Supplementary file 1 — Additional file 1. Survey OTAPA. [file 13195_2023_1190_MOESM1_ESM.pdf]

Online survey to your experiences of clinical trial participation. Thank you for participation in this research.

Short overview of this research
When designing clinical trials to Alzheimer's disease, it is important to consider the wishes and preferences of participants and their study partners. Our research focuses on the wishes and motivations of participants in clinical trials. Little is known about motivations, facilitators and barriers of participation in a clinical trial and the preferences of the participants regarding trial design.

You have participated in a clinical trial at the Brain Research Center, and for our study we are interested in your experiences. How do you look back on you trial participation? What is your advice for trial designers? We will also address your preferences in designing a future trial. For example, you should think of how often you want to come to the center or the maximum number of brain scans you would like to undergo in a clinical trial.

Aim of the study
The purpose of this study is to find out the motivations for participating in a clinical trial and to gain in- depth sight into your vision and wishes in designing a clinical trial to Alzheimer's disease.
The results of this research may lead to recommendation for designing future clinical trials to Alzheimer's disease.

Survey
You are participating or have participated in a clinical trial at the Brain Research Center. This list contains questions about how you experienced your participation. Your answers will be used in scientific research that aims to improve future studies. The survey is part of a study carried out by researchers from the Alzheimer Center Amsterdam in Amsterdam UMC, location VUmc. Your data will be anonymized.

Your records
All your details remain confidential. Only the researcher knows which number you have. The research data cannot be traced back to you when published in a scientific journal. We will save the data for 15 years. After that, the data will be destroyed.

More information about your rights when processing data
For more information about your rights when processing your personal data, please contact Lois Ottenhoff via l.ottenhoff@amsterdamumc.nl. Lois Ottenhoff is responsible for following the rules of processing personal data.

Survey instructions

- The survey will take about 20-30 minutes
- Read each question completely before choosing an answer
- Choose the answer that suits you best
- If you are allowed to choose more than one answer, this will be mentioned within the question
- Some questions seems ‘ duplicate’, but we ask you to complete them anyway. They are meant to look at you situation differently.

By completing this questionnaire, you consent to the use of your data for scientific research.
Do you want to continue?

- ☐ Yes
- ☐ No

General information

1. Are you a participant or study partner?
    - ☐ Participant
    - ☐ Study partner

2. Did someone help you fill in this questionnaire?
    - ☐ No, I just filled in the list.
    - ☐ Yes, someone helped me fill in the list

3. Are you currently in a clinical trial?
    - ☐ Yes
    - ☐ No

4. Enter your date of birth here

    ______-______-______

5. What is your gender?
    - ☐ Male
    - ☐ Female

6. What is the highest level of education you have completed?
    - ☐ Less than 6 classes of primary school
    - ☐ 6 classes of primary school
    - ☐ More than primary school / primary school without further completed education
    - ☐ Secondary school
    - ☐ Secondary vocational education
    - ☐ University / higher education

7. Do you have an Alzheimer's diagnosis?
    - ☐ Alzheimer's dementia
    - ☐ Mild Cognitive Impairment due to Alzheimer's disease
    - ☐ No diagnosis, but carrier of gene and proven Alzheimer's protein present (Amyloid-Beta)
    - ☐ No diagnosis

8. How many trials at the Brain Research Center have you participated in? (if you are now participating in a trial, you may count this)
    - ☐ 1 trial
    - ☐ 2 trials
    - ☐ 3 trials
    - ☐ 4 trials
    - ☐ 5 trials
    - ☐ More than 5 trials

9. Which trial(s) did you participate in?

……………………………..

If you have participated in several trials, you may answer the following questions about the last trial in which you participated.

10. Have you completed/completed the entire trial?
    - ☐ Yes
    - ☐ No
    - ☐ I don't know

11. Did you stop your trial yourself?
    - ☐ Yes
    - ☐ No
    - ☐ Don't know

12. Why did you stop participating?

    ……………………………..

13. Was the trial stopped by Brain Research Center?
    - ☐ Yes
    - ☐ No
    - ☐ Don't know

Clinical trial participation

1. What was the main reason you signed up for your first trial at the Brain Research Center? Below are frequently mentioned reasons of participants to participate in a trial. Please tick which one applies to you. You may select multiple options.

   - ☐ For the future generation
   - ☐ This way I will receive better care
   - ☐ For science
   - ☐ I think this is the best treatment
   - ☐ My doctor recommended it
   - ☐ I think I am better monitored
   - ☐ I find it interesting
   - ☐ Gives a useful way to fill my day

2. Did you have any other reasons to sign up?

   ………………………………………

3. Did you have a good idea in advance of what the trial would look like/did you know what you were getting into?

   ……………………………………..

4. How satisfied are you with your participation?
   (1= not satisfied, 2= slightly satisfied, 3= satisfied, 4= very satisfied, 5= extremely satisfied)

5. Did you regret your participation?
   (1= never, 2= rarely, 3= sometimes, 4 = often, 5= very often)

6. Have you ever thought to stop participating?
   (1= never, 2= rarely, 3= sometimes, 4 = often, 5= very often)

7. Why did you want to stop?

   ……………………………………..

8. Would you participate in a new trial again?
   - ☐ Yes
   - ☐ No

9. Why?

……………………………………..

10. Would you recommend participating in an Alzheimer's disease drug trial to others?
    - ☐ Yes
    - ☐ No

11. Why/why not?

……………………………………..

12. What was the most positive aspect of your participation?

……………………………………..

13. What was the most negative aspect of your participation?

……………………………………..

Trial features

Now there are a number of questions about different features that must be completed in each trial. We ask you how stressful you have experienced these parts, think of brain scans or memory tests.

1. What did you find the most high burden part of the trial These are answer options that are often mentioned by participants. You may select multiple options.
   - ☐ Visit to the center
   - ☐ Blood sampling
   - ☐ ECG
   - ☐ Physical examination
   - ☐ Memory tasks
   - ☐ Questionnaires about sadness, quality of life and daily functioning
   - ☐ Lumbar puncture
   - ☐ Brain scan/MRI scan
   - ☐ Brain scan with IV/PET scan

2. What did you find the least burdensome part of the trial? These are answer options that are often mentioned by participants. You may select multiple options.
   - ☐ Visit to the center
   - ☐ Blood sampling
   - ☐ ECG
   - ☐ Physical examination
   - ☐ Memory tasks
   - ☐ Questionnaires about sadness, quality of life and daily functioning
   - ☐ Lumbar puncture
   - ☐ Brain scan/MRI scan
   - ☐ Brain scan with IV/PET scan

The purpose of the following questions is that you indicate how stressful you experienced this part of the trial on a scale from 0 (not stressful) to 100 (very stressful).

1. How burdensome have you experienced the **number of visits to the center**?

Not burdensome |—|—|—|—|—|—|—|—|—|—| high burdensome

0 50 100

2. How burdensome have you experienced the **brain scan/MRI-scan**?

Not burdensome |—|—|—|—|—|—|—|—|—|—| high burdensome

0 50 100

3. How burdensome have you experienced the **lumbar puncture**?

Not burdensome 0 50 100 high burdensome

4. How burdensome have you experienced the **PET scan**?

Not burdensome 0 50 100 high burdensome

5. How burdensome have you experienced the **memory assessments**?

Not burdensome 0 50 100 high burdensome

6. How burdensome have you experienced the **depression questionnaires**?

Not burdensome 0 50 100 high burdensome

7. How burdensome have you experienced the **quality of life questionnaires**?

Not burdensome 0 50 100 high burdensome

8. How burdensome have you experienced the **physical examination by the doctor**?

Not burdensome 0 50 100 high burdensome

9. How burdensome have you experienced the **blood test**?

Not burdensome 0 50 100 high burdensome

10. How burdensome have you experienced the **ECG heart rate**?

Not burdensome 0 50 100 high burdensome

Frequency of trial visits

Now there are again a number of questions about different parts of a trial. The intention is that you indicate the maximum number of visits you would like to undergo for each part.

1. A trial usually lasts between 6 months and 5 years.
   What would be the **best trial duration** for you?

years

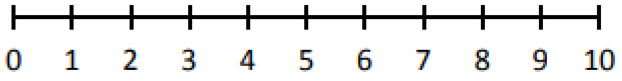

2. How often would you like to **visit the research center**?
   - ☐ Once a week
   - ☐ Once a month
   - ☐ Once every three months
   - ☐ Once every six months
   - ☐ Once a year

3. How often would you like to undergo a **brain scan/MRI scan**?
   - ☐ Once a week
   - ☐ Once a month
   - ☐ Once every three months
   - ☐ Once every six months
   - ☐ Once a year

4. How often would you like to undergo a **lumbar puncture**?
   - ☐ Once a week
   - ☐ Once a month
   - ☐ Once every three months
   - ☐ Once every six months
   - ☐ Once a year

5. How often would you like to undergo a **brain scan with IV/PET-scan**?
   - ☐ Once a week
   - ☐ Once a month
   - ☐ Once every three months
   - ☐ Once every six months
   - ☐ Once a year

6. How often would you like to undergo the **memory assessments**?
   - ☐ Once a week
   - ☐ Once a month
   - ☐ Once every three months
   - ☐ Once every six months
   - ☐ Once a year

7. How often would you like to undergo the **depression questionnaires**?
   - ☐ Once a week
   - ☐ Once a month
   - ☐ Once every three months
   - ☐ Once every six months
   - ☐ Once a year

8. How often would you like to undergo the **quality of life questionnaires**?
   - ☐ Once a week
   - ☐ Once a month
   - ☐ Once every three months
   - ☐ Once every six months
   - ☐ Once a year

9. How often would you like to undergo the **daily functioning (IADL) questionnaires**?
   - ☐ Once a week
   - ☐ Once a month
   - ☐ Once every three months
   - ☐ Once every six months
   - ☐ Once a year

10. How often would you like to undergo the **physical/neurological assessments**?
    - ☐ Once a week
    - ☐ Once a month
    - ☐ Once every three months
    - ☐ Once every six months
    - ☐ Once a year

11. How often would you like to undergo the **blood test**?
    - ☐ Once a week
    - ☐ Once a month
    - ☐ Once every three months
    - ☐ Once every six months
    - ☐ Once a year

12. How often would you like to undergo an **ECG heart rate**?
    - ☐ Once a week
    - ☐ Once a month
    - ☐ Once every three months
    - ☐ Once every six months
    - ☐ Once a year

11. Are there parts of the trial that you would like to undergo ''as little as possible'' or ''not at all'' ? Would you like to explain what is ''as little as possible'' for you?

    ………………………………….

Motivations for participating in a clinical trial

The following items deal with a number of factors that are important when considering participating in a trial.

1. What would make participation easier for you. These are answer options that are often mentioned by participants. Several answer options are possible.
    - ☐ Shorter travel time
    - ☐ Home visits
    - ☐ Less often a visit to the center
    - ☐ Less often an lumbar puncture
    - ☐ Less often a brain scan
    - ☐ Less often memory tests
    - ☐ Less often depression questionnaires
    - ☐ Less chance of placebo
    - ☐ No study partner
    - ☐ More reimbursement
    - ☐ Less often brain scan with IV / PET scan

2. What do you think is the most important factor in participating in a trial? These are answer options that are often mentioned by participants. Several answer options are possible.
    - ☐ Chance of side effects
    - ☐ Distance to the center
    - ☐ Keep the same doctor/neuropsychologist/study coordinator throughout the trial
    - ☐ Chance of placebo
    - ☐ Number of visits per month
    - ☐ Time of visit
    - ☐ Reputation of the research center
    - ☐ Being able to participate in a new trial after completing a trial
    - ☐ Privacy
    - ☐ Getting paid
    - ☐ Feedback from the trial
    - ☐ Feedback of your own results in the trial

3. What do you think is the least important factor for taking part in a trial? These are answer options that are often mentioned by participants. Several answer options are possible.

    - ☐ Chance of side effects
    - ☐ Distance to the center
    - ☐ Keep the same doctor/neuropsychologist/study coordinator throughout the trial
    - ☐ Chance of placebo

- ☐ Number of visits per month
- ☐ Time of visit
- ☐ Reputation of the research center
- ☐ Have the ability to participate in a new trial after completing a trial
- ☐ Privacy
- ☐ Getting paid
- ☐ Feedback from the trial
- ☐ Feedback on your own results in the trial

4. Personal results of the trial are usually not shared. How important do you think that these results are shared with the participants (such as results of the memory tasks or MRI scan)?

   - ☐ Very important
   - ☐ Slightly important
   - ☐ Neutral
   - ☐ Not important
   - ☐ Not important at all

5. The results of the trial are usually only announced much later. How important do you think that the results of the research are shared with the participants?

   - ☐ Very important
   - ☐ Somewhat important
   - ☐ Neutral
   - ☐ Not important
   - ☐ Not important at all

6. Why do you think some people don't want to participate in a trial?

   …………………………………….

7. What would help to participate in a trial?

   ……………………………………

Conclusion

If you have any comments, please write them below:

…………………………………….

……………………………………..

……………………………………..

Thank you for completing the questionnaire.

Follow-up research
Based on the results of this questionnaire, we will go into more detail about wishes, motivations and experiences of participants in a trial. We will also elaborate on your preferences in shaping a future trial and your advice regarding trial design.

To this end, we will organize various meetings to discuss this with participants.

This will take place in the Amsterdam UMC and will take approximately 2 hours.

May we approach you for this:

- ☐ Yes
- ☐ No
